# SMA-EUROPE workshop report: opportunities and challenges in developing clinical trials for spinal muscular atrophy in Europe

**DOI:** 10.1186/1750-1172-8-44

**Published:** 2013-03-20

**Authors:** Nathalie Kayadjanian, Arthur Burghes, Richard S Finkel, Eugenio Mercuri, Francoise Rouault, Inge Schwersenz, Kevin Talbot

**Affiliations:** 1 TransBioMed solutions LLC, Murray Hill, USA; 2 College of Medicine, Ohio State University, Columbus, USA; 3 Nemours Children’s Hospital, Orlando, USA; 4 Pediatric Neurology Unit, Catholic University, Rome, Italy; 5 Association Française contre les Myopathies, Evry, France; 6 SMA-EUROPE, Freiburg, Germany; 7 Nuffield Department of Clinical Neurosciences, Oxford University, Oxford, UK

**Keywords:** Spinal muscular atrophy, Clinical trials, Preclinical drug selection, Phase III trial planning, Patient registries, Neonatal trials, Newborn screening, Standards of care, European regulations, Efficacy biomarkers

## Abstract

Spinal muscular atrophy (SMA) is the most common lethal recessive disease in childhood, and there is currently no effective treatment to halt disease progression. The translation of scientific advances into effective therapies is hampered by major roadblocks in clinical trials, including the complex regulatory environment in Europe, variations in standards of care, patient ascertainment and enrolment, a narrow therapeutic window and a lack of biomarkers of efficacy. In this context, SMA-Europe organized its first international workshop in July 2012 in Rome, gathering 34 scientists, clinicians and representatives of patient organizations to establish recommendations for improving clinical trials for SMA^a^.

## Correspondence

SMA is caused by homozygous absence of the SMN1 gene and retention of the SMN2 gene [1] resulting in degeneration of spinal motoneurons. The phenotypic spectrum ranges from severe (Type I) to mild (Type III, IV). To date, 26 SMA clinical trials of 12 different drugs have failed to show any significant effect on disease progression [2].

Analyses of past clinical trials show issues in the drug selection process and the study design. Drug selection based on preclinical data obtained from transformed cell lines may not be a reliable predictor of a therapeutic effect in motoneurons. The majority of Phase I and II trials have used patients with Types II and III SMA and Phase III trial design is underdeveloped.

Trial implementation in Europe faces specific challenges. Information on the SMA population is fragmentary, reliable natural history data is lacking and the degree of variation in incidence and survival between countries is unknown. SOC vary from center to center [3] making the creation of homogeneous trial populations difficult. The lack of a homogeneous regulatory framework between individual European member States leads to delays and increased costs.

SMA Type I is estimated to constitute 60% of the total incident SMA population with SMA Types II and III comprising 30% and 10% respectively [4]. Analyses of patient registries (Table 1) show that SMA Type I is underrepresented, suggesting that a significant proportion of SMA cases go unrecorded. Given the short life expectancy of SMA Type I patients and the narrow therapeutic time-window, it is vital to reduce the age of diagnosis and time for recording into registry.

**Table 1:** SMA patient registries

**Database information**	**Families of SMA**	**International IU FSMA Registry**	**MDA**	**Treat-NMD**	**Estimated**
Number of patients	11,000	3,000	5,500	2,400	
SMA subtypes (%)	I* 51	I 28	I 19.1	I 20	I 60
	II* 24	II 33.5	II 35.4	II 43	II 30
	III* 12	III 26	III 28.8	III 37	III 10
	IV/Unknown* 13		IV 16.7		
Average age of diagnosis (months)	I 4.9				
	II 18.7				
Average age at entry into registry (months)	I 6				
	II 20				

*Calculated from approximately 1,000 newly diagnosed patients contacting FSMA over the previous three-year period (Courtesy of Families of SMA).

Treatment in late-stage of the disease could partly explain past trial failures. Mouse studies show that neonatal treatment is probably critical for efficacy [5-7]. Clinical studies (Carnival-Type I, STOPSMA) suggest that the first few months of life are the most critical times of denervation in SMA Types I and II. These findings support the implementation of neonatal trials and the development of strategies for identifying patients at pre-symptomatic or early-symptomatic stages.

Several arguments support newborn screening (NBS) for SMA despite the lack of treatment^b^. SMA is an early onset, often fatal disease with a well-understood molecular genetic defect. Early diagnosis and proactive care extend significantly the lifespan of SMA patients, especially for SMA Type I [8]. Treatment at the neonatal stage could be effective. Advances in genomic technologies now make NBS feasible. While pilot studies exploring NBS for SMA are underway in the US, discussion about implementing NBS in Europe is in its infancy.

Several issues should be considered before conducting clinical trials in neonates. A key concern is to define whether the use of placebo controls or natural history data should be preferred in clinical trials. Natural history data in the immediate postnatal period is lacking. Another challenge is the absence of validated markers of disease progression. For SMA Type I, electrophysiological measures such as MUNE and CMAP may be better indicators of efficacy than survival [9]. Biomarkers like ventilatory function and muscle mass quantification should be explored during the postnatal period. Treatment of pre-symptomatic infants also raises ethical issues. In determining trial duration, efficacy should be balanced with the costs and family burden. Finally, the feasibility of establishing consensus guidelines for SOC for neonates should be explored. In parallel, the effect of SOC on improving survival and on the therapeutic window should be assessed.

Biomarkers of treatment effects which are clinically meaningful and sensitive to disease changes within the time-frame of trials are urgently needed. Currently, numerous overlapping motor functional scales serve as clinical end-points. Although ongoing Rasch analyses^c^ should help to create less redundant and more robust scales, the field would benefit from developing scales that are multifactorial, linear, clinically relevant and sensitive to clinical change. These should be adapted for pre-symptomatic, early-symptomatic and chronic stages for each SMA subtype. Consistent with most therapeutic approaches intended to increase SMN levels, efforts to develop surrogate markers have focused on measuring SMN expression in blood. Whether this reflects what is happening in relevant nervous system tissues remains uncertain, other biomarkers need to be tested^d^ or developed.

### Summary of recommendations

1. The preclinical effectiveness of therapeutics should be tested in SMA animal models.

2. Plan the feasibility of ensuing phase I, II and III clinical trials.

3. Harmonization of the European regulatory process for drug approval should be strongly advocated.

4. Methods should be developed to either ensure patients are drawn from populations with uniform SOC or for integrating populations with different SOC within one clinical trial.

5. The optimal therapeutic window should be targeted in SMA clinical trials by:

Reducing age at enrollment into clinical trials.

Advancing early diagnosis (NBS).

Reducing time for entry into registry.

Enrolling pre-symptomatic siblings.

6. Specific issues related to SMA Type I patients should be addressed:

Increase registry enrollment

Explore SOC guidelines

Study natural history of the early postnatal period.

Develop new biomarkers.

7. Biomarkers

Develop measures of upper limb function for non-ambulant patients.

Explore muscle biomarkers (MUNE/CMAP, muscle MRI, EIM).

Test the SMA Foundation’s biomarker panel^d^.

## Endnotes

^a^More information can be found on the SMA-EUROPE website.

^b^Most NBS programs are based on the criteria defined by Wilson and Jungner in 1968. With the advances of genetic technology over the past forty years, the original ten principles laid out by Wilson and Jungner have been revised [10]. For example, the criterion “treatment available” is often broadened in many discussions to include broader health benefits and other advantages to parents, especially avoiding a diagnostic odyssey and informed reproductive choice [11].

^c^Rasch analysis is supported by the patient advocacy group of the International Coordinating Committee for SMA clinical trials which includes families of SMA, Fight SMA, MDA , the SMA Foundation and SMA-Europe.

^d^As a result of the BforSMA study discovery effort, a panel of 27 validated plasma protein biomarkers was created by Myriad RBM in collaboration with the SMA Foundation. The SMA-MAP panel is available at Myriad RBM.

## Abbreviations

CMAP: Compound muscle action potential; EIM: Electrical impedance myography; MRI: Magnetic resonance imaging; MUNE: Motor unit number estimation; SMN: Survival motor neuron; SOC: Standards of care.

## Competing interests

The authors declare no financial or non-financial competing interest.

## Authors’ contributions

All the listed authors: 1) have made substantial contributions to the conception, organization of the workshop and design of this letter; 2) have been involved in drafting the manuscript or revising it critically for important intellectual content; and 3) have given final approval of the version to be published.

## Authors’ information

NK is involved in translational biomedical research for SMA. AB and KT are involved in basic and translational research on SMA. RF, EM are involved the development of outcome measures and the design and execution of clinical trials in SMA. FR is involved in the international scientific management of SMA-EUROPE. IS represents the voice of SMA patients in Germany and is vice-president of SMA-EUROPE.
